# Effects of Tobacco Versus Electronic Cigarette Usage on Nonsuicidal Self-Injury and Suicidality Among Chinese Youth: Cross-Sectional Self-Report Survey Study

**DOI:** 10.2196/47058

**Published:** 2023-07-07

**Authors:** Yinzhe Wang, Shicun Xu, Xiaoqian Zhang, Yanwen Zhang, Yi Feng, Yuanyuan Wang, Runsen Chen

**Affiliations:** 1 Department of Human Development & Quantitative Methods Graduate School of Education University of Pennsylvania Philadelphia, PA United States; 2 Vanke School of Public Health Tsinghua University Beijing China; 3 Northeast Asian Research Center Jilin University Changchun, Jilin China; 4 China Center for Aging Studies and Social-Economic Development Jilin University Changchun, Jilin China; 5 Department of Psychiatry Tsinghua University Yuquan Hospital Beijing China; 6 Mental Health Center Central University of Finance and Economics Beijing China; 7 Psychology Application Center for Enterprise and Society School of Sociology and Psychology Central University of Finance and Economics Beijing China; 8 Key Laboratory of Brain Cognition and Education Sciences Ministry of Education Beijing China; 9 Institute for Healthy China Tsinghua University Beijing China

**Keywords:** electronic cigarettes, tobacco, conventional cigarettes, SGM, nonsuicidal self-injury, suicidality, suicidal, cigarette, cigarettes, suicide, self-harm, mental health, sexual minority, gender minority, sexual and gender minority, transgender, youth, cross-sectional, survey, smoker, smoking

## Abstract

**Background:**

The increase in tobacco/conventional cigarette (CC) and electronic cigarette (EC) usage among Chinese youth has become a growing public health concern. This is the first large-scale study to compare the impact of CC and EC usage on risk for nonsuicidal self-injury (NSSI) and suicidality in cis-heterosexual and sexual and gender minority (SGM) youth populations in China.

**Objective:**

This study examines the CC and EC risks for NSSI and suicidality among Chinese youth and compares the extent to which SGM and cis-heterosexual youth’s risks for NSSI and suicidality are influenced by their CC and EC usage and dependence.

**Methods:**

A total of 89,342 Chinese participants completed a cross-sectional self-report survey in 2021. Sociodemographic information, sexual orientations, gender identities, CC and EC usage, CC and EC dependence, and risks for suicidality and NSSI were assessed. The Mann-Whitney *U* test and chi-square test were performed for nonnormally distributed continuous variables and categorical variables, respectively. The multivariable linear regression model was used to examine both the influence of CC and EC usage and CC and EC dependence on NSSI and suicidality as well as the interaction effects of CC and EC usage and CC and EC dependence on NSSI and suicidality by group.

**Results:**

The prevalence of CC usage (*P*<.001) and dependence (*P*<.001) among SGM participants was lower than that among their cis-heterosexual counterparts. However, the prevalence of EC usage (*P*=.03) and EC dependence (*P*<.001) among SGM participants was higher than that among their cis-heterosexual counterparts. The multivariable linear regression model showed that CC dependence and EC dependence had a unique effect on NSSI and suicidality (CCs: B=0.02, *P*<.001; B=0.09, *P*<.001; ECs: B=0.05, *P*<.001; B=0.14, *P*<.001, respectively). The interaction effects of (1) CC usage and group type on NSSI and suicidality (B=0.34, *P*<.001; B=0.24, *P*=.03, respectively) and dual usage and group type on NSSI and suicidality (B=0.54, *P*<.001; B=0.84, *P*<.001, respectively) were significant, (2) CC dependence and group type on NSSI were significant (B=0.07, *P*<.001), and (3) EC dependence and group type on NSSI and suicidality were significant (B=0.04, *P*<.001; B=0.09, *P*<.001, respectively). No significant interaction effect was observed between EC usage and group type on NSSI and suicidality (B=0.15, *P*=.12; B=0.33, *P*=.32, respectively) and between CC dependence and group type on suicidality (B=–0.01, *P*=.72).

**Conclusions:**

Our study shows evidence of intergroup differences in NSSI and suicidality risks between SGM and cis-heterosexual youth related to CC and EC usage. These findings contribute to the growing literature on CC and EC in cis-heterosexual and SGM populations. Concerted efforts are necessary at a societal level to curb the aggressive marketing strategies of the EC industry and media coverage and to maximize the impact of educational campaigns on EC prevention and intervention among the youth population.

## Introduction

Electronic cigarettes or e-cigarettes (ECs) are battery-based vaping devices simulating tobacco/conventional cigarette (CC) smoking by aerosolizing artificially manufactured liquid solutions containing nicotine for users’ inhalation [1]. The association between the usage of CCs and suicidality is well-established, with previous studies showing that regular usage of CCs is associated with an increased risk of suicidality (ie, suicidal ideation, plan, and attempt) [2-4]. Several studies have reported the neurotoxicity of CC usage on adolescents’ brain structure, increasing the risk of oxidative stress–related neurological disorders while promoting emotional dysregulation [4-6]. Subsequently, the emotional dysregulation brought about by CC usage could further aggravate individuals’ pre-existing mental health conditions (eg, depressive thinking featured by hopelessness and worthlessness). Combined with relevant social factors (eg, isolation), the complex interplay between CC usage and mental health risks may heighten the risk of suicidality among CC users [4-6].

Similar to CC usage, EC usage can impose not only physical risks (ie, nicotine dependence, cardiovascular injury, respiratory system damage, and noncancer pulmonary disease) but also mental health threats, including but not limited to depression, anxiety, and nonsuicidal self-injury (NSSI) [5,6]. In addition, recent studies suggest that nicotine usage, including CC and EC usage, has a bivariate relationship with NSSI, underscoring the potential relationship between NSSI and both CC and EC usage [7,8]. Nevertheless, although findings from the abovementioned studies have shown the impact of CC and EC usage on youth NSSI and suicidality, their differences in chemical composition, method of consumption, and sociocultural perceptions are drastically different from each other. First, regarding the chemical composition, compared to tobacco in CC, EC is featured with a liquid containing both nicotine and flavoring, with a high percentage of it being glycerol or propylene glycerol [9]. Second, concerning the method of consumption, compared to the lighting process of CC, EC often uses a power button to activate an atomizer to generate an aerosol from its liquid, which simulates smoke from CC and requires no fire and burning, thereby making its usage more prevalent in places where CC is banned [9,10]. Third, with regard to sociocultural perceptions, EC has been marketed as less harmful than CC in the Chinese market, with relatively limited regulations on the minimum age to purchase compared to CC [10]. Due to the abovementioned differences, EC and CC may influence youth NSSI and suicidality differently.

Findings from past research postulate that college students’ high susceptibility to mental health risks, exemplified in forms of anxiety, depression, and stress, when facing concerns resulting from sudden environmental changes (ie, moving from one’s hometown to another city), social isolation, social alienation, worries of financial independence, high academic performance expectations, and learning style change corresponds to their high risk of suicidality [11,12]. Previous research argues that college students often begin to explore their gender and sexual identity in their college lives, during which the disparity between cis-heterosexual and sexual and gender minority (SGM) youth in suicidality may surface [13,14]. The 2016 National Health Interview Survey showed that 20.5% of sexual minority young adults smoke CCs, which is much higher than that reported among their heterosexual counterparts (ie, 15.3%) [15]. The prevalence of CC usage in the transgender and gender-diverse population is also higher than that in their cisgender counterparts [15,16]. Similarly, recent studies have also found that the prevalence of EC usage in the SGM population is higher than that in their cis-heterosexual counterpart [17-20]. Specifically, there is burgeoning evidence from previous studies that suggest SGM youth’s current usage (SGM: 9.4%; cis-hetero: 4.9%), lifetime usage (SGM: 25.1%; cis-hetero: 14.3%), and past 30-day usage of ECs (SGM: 28.5%; cis-hetero: 23.4%) are all higher than those among cis-heterosexual youth, indicating this group’s vulnerability toward EC usage [18-20].

Such a disparity in EC and CC usage between SGM and cis-heterosexual youth could be potentially explained by the minority stress model. Minority stressors, including but not limited to heterosexist discrimination, social rejection, social isolation, and homo/bi/transphobic harassment, could further worsen the stigmatization of SGM youth’s identities while inducing higher stress levels among them, contributing to their increased risk of substance usage [21]. Furthermore, besides the minority stress experienced by SGM youth, the tobacco industry has further exploited their vulnerability since they have historically been targeting SGM youth as a major consumer crowd and pushed forward relevant campaigns such as the Project Subculture Urban Marketing for gay individuals in San Francisco [22]. Nevertheless, to date, no study has examined the relationship between the usage of CCs and ECs on college students’ NSSI and suicidality while investigating potential intergroup differences existing between cis-heterosexual and SGM youth, leaving a critical gap in the literature. Most of the current CC and EC research [4-22] examining the increasing risks of youth for NSSI and suicidality focused on studying CC and EC usage separately and did not further investigate the different extents to which the SGM and cis-heterosexual youth’s risks for NSSI and suicidality were affected by CC and EC usage, ignoring the potentially existing intergroup differences. Our cross-sectional study aims to fill this gap by investigating the effects of CC and EC on NSSI and suicidality and the difference in the effects between SGM and cis-heterosexual youth. This study’s hypotheses postulate that (1) cigarette usage (including CC and EC usage) and cigarette dependence (including CC and EC dependence) have a unique and positive impact on both NSSI and suicidality, (2) cigarette usage has a more pronounced impact on NSSI and suicidality among SGM youth than on NSSI and suicidality among their cis-heterosexual peers, and (3) cigarette dependence has a more severe impact on NSSI and suicidality among SGM youth than on NSSI and suicidality among their cis-heterosexual peers.

## Methods

### Participant Recruitment

University students from 63 universities and colleges in the Jilin province, China, were invited to participate in a cross-sectional web-based self-report survey. Recruitment was performed via universities’ and colleges’ staff teachers and targeted WeChat groups (similar to WhatsApp groups). Students who were interested in participation entered the secure and anonymous questionnaire through the QR code mentioned in the study poster that was distributed by school staff teachers. Students were first asked to thoroughly read the study description, and those who selected the commensurate button and continued to answer the questionnaire were deemed as consented to this study. Individuals who successfully completed this questionnaire were offered the opportunity to join a cash prize lottery by accessing a separate link. The description of the cash prize lottery was listed on the front page of the web-based survey, and the amount was carefully calibrated to strike a balance between being sufficient to encourage participation and minimize dropout without being excessively large to create an undue influence on participants’ behavior or reporting. Respondents’ data were collected between October 26 and November 18, 2021, via Credamo [23].

For statistical quality control of this study, we excluded respondents who (1) were younger than 15 years (ie, below the lowest threshold of the regular age range of college or university students in China), (2) failed 2 or more of the 4 attention check questions (ie, this question is an attention check question. There are many colors of the sky, which can be green, blue, red and black, but, for this question, please choose “green” for your answer) that were designed to assess whether survey respondents were focused during their responding process, and (3) showed presence of logical contradictions or inconsistencies within responses (eg, male participants choosing “male” for sexual preference while choosing “heterosexual” for sexual orientation), omission of answers (eg, leaving blanks in their answers instead of choosing options such as “none of the above” or “prefer not to say”), or their answers showed evidence of patterned or nondiscriminatory responses (eg, consistently choosing the first option across multiple questions). The final sample consisted of 89,342 participants, among which 8853 were SGM respondents and 80,489 were cis-heterosexual respondents.

### Study Measures

#### Gender Identity

Participants’ sociodemographic information, including age, ethnicity (Han ethnicity or non-Han ethnicity), educational background (undergraduate, master, or doctoral students), residence areas prior to university/college enrollment (urban or rural), only-child status, and annual family income, was collected. This study’s 3 gender identity assessment questions were based on the GenIUSS published report from the Williams Institute, University of California, Los Angeles [24]. First, participants were asked, “What was your sex assigned at birth? (gender specified at birth or the one listed on your original birth certificate)” with options being male and female. Second, participants were asked, “What is your gender identity? (your personal thoughts and understanding of your own gender)” with options being cisgender, transgender, nonbinary/gender nonconforming/agender/others, unsure, and cannot understand the question. Third, participants were asked, “If you can only choose from 1 option below, which gender option best describes you?” with options being male, female, transgender male (female-to-male or longing to become male), transgender female (male-to-female or longing to become female), genderqueer/nonbinary/gender nonconforming, cross-dresser, unsure, and not listed (please write down your answer in the blank). The third question served the function of discerning self-identified transgender male and female participants in our sample, including male-to-female, female-to-male, and participants who desired to live their lives as the opposite sex. Written answers from participants who chose “not listed (please write down your answer in the blank)” were manually inspected and reviewed by 2 research assistants who then recoded those participants’ gender identities after reaching a consensus in thorough discussions. Due to the sociocultural unfamiliarity with the gender identity glossary, a validity check was performed for the first and third questions. Transgender and gender nonconforming participants, including transgender males, transgender females, and participants who were genderqueer/nonbinary/gender nonconforming, who answered consistently across all 3 questions were included in the analyses.

#### Sexual Orientation

This study’s 3 sexual orientation assessment questions were also based on the SMART published report from the Williams Institute, University of California, Los Angeles [25]. First, participants were asked, “Which one of the following best describes your sexual orientation?” with choices being asexual (not sexually attracted to others or have low interest in sexual activity), homosexual, heterosexual, bisexual (can be romantically and sexually attracted to both males and females), pansexual (can be romantically and sexually attracted to anyone, regardless of their sex or gender identity), and not listed. Participants were then asked to answer the second and third questions, “What kind of person are you sexually attracted to?” and “What kind of person are you romantically attracted to?” with the same set of choices ranging from male, female, both male and female, any gender (including transgender, genderqueer, and nonbinary), unsure, and not listed (please write down your answer in the blank). For participants who chose “not listed” for the first question, 2 aforementioned research assistants manually inspected their written answers, confirmed their answers’ consistency by comparing their answers to the second and third questions, and eventually recoded their sexual orientations after reaching consensus in thorough discussions. Participants who sexually identified as homosexual, bisexual, and pansexual were included in the final analyses. Upon coding completion, all participants in this study were successfully labeled as SGM and cis-heterosexual.

#### CC and EC Usage

We employed 2 screening questions to assess participants’ CC and EC usage. Participants were asked to answer the first question, “Do you currently have smoking habits?” with options including yes and no. Those who chose yes continued to answer the remaining questions related to CC and EC usage, while others directly skipped to the next section of the questionnaire. Those who chose yes were then asked the second question, “Do you usually smoke e-cigarettes or conventional cigarettes?” with options including e-cigarettes, conventional cigarettes, and both. First, those who chose conventional cigarettes were then assessed by answering the 6-item validated Chinese version of the Fagerström Test of Nicotine Dependence (FTND) [26,27]. Their scores, ranging from 0 to 10, were calculated by summing up their responses, with higher scores indicating higher levels of nicotine dependence. Scores from 0 to 2 demonstrate very low dependence; scores from 3 to 4 demonstrate low dependence; scores at 5 demonstrate medium dependence; scores from 6 to 7 demonstrate high dependence; and scores from 8 to 10 demonstrate very high dependence. The validated Chinese version has been used in previous research for similar purposes, demonstrating good internal consistency and validity [28,29]. Second, for those who chose ECs, we revised the validated Chinese version of the FTND to measure their EC dependence. Although the revised scale also contains 6 items, we expanded the measurement ranges of both the first and fourth items by adding 2 additional options for each item, increasing the score range from 0-10 to 0-14. Nevertheless, participants still scored on a response scale, with a higher score indicating a higher level of EC dependence. Third, those who chose “Both” were asked to answer both sets of questions. For those who did not answer the corresponding part of the questionnaire, scores were set to 0. In this study, the reliability values of FTND (ω=0.83) and e-FTND (ω=0.80) were acceptable.

#### Suicidality

The Suicidal Behaviors Questionnaire-Revised (SBQ-R) was designed to measure individuals’ suicide-related behaviors from 4 different dimensions, that is, individuals’ history of suicide attempt(s), individuals’ frequency of suicidal ideation, individuals’ suicide threats, and individuals’ future suicide attempts likelihood [30]. The validated Chinese version of the SBQ-R contains 4 items, with the total score ranging from 3 to 18 and higher scores indicating higher levels of suicidality [31]. A total score of 7 or higher was considered a high suicide risk, while a total score below 7 was considered a moderate or low suicide risk. In this study, the ω of SBQ-R was 0.81.

#### NSSI Analysis

Two items adapted based on the Clinician-Rated Severity of Nonsuicidal Self-Injury were used in this study [32]. The first item asked participants whether they have ever engaged in NSSI, with options including “Yes. I engaged in NSSI more than one year ago,” “Yes, I engaged in NSSI within the past year,” and “no.” The second item asked how many days during the past year did participants engage in NSSI, with options including none, 1 to 4 days, 5 to 7 days, 8 to 11 days, and 12 days or more. We combined these 2 items as 1 item for participants to answer, with options ranging from “None,” “I engaged in NSSI more than one year ago,” “I have had NSSI 1 to 4 days in the past year,” “I have had NSSI 5 to 7 days in the past year,” “I have had NSSI 8 to 11 days in the past year,” to “I have had NSSI 12 days or more in the past year,” with participants’ scores ranging from 0 (none) to 6 (12 days or more in the past year). Since NSSI for 5 or more days in the past year was the clinically recommended cutoff, as suggested by the American Psychiatric Association [32], participants in this study were classified into 3 groups (ie, group with no NSSI history, group with less than 5 days of NSSI within the past year, and group with more than 5 days of NSSI within the past year) for further analyses.

### Data Analysis

First, we performed a descriptive analysis of the sociodemographic variables of the SGM and the cis-heterosexual groups. The Mann-Whitney *U* test and chi-square test were performed for nonnormally distributed continuous variables (eg, scores of CC dependence, EC dependence, NSSI, suicidality) and categorical variables (eg, classifications of cigarette usage, NSSI, and suicidality), respectively. Our primary analyses using the multivariable linear regression model involved examinations of both the influence of CC usage and EC usage on NSSI and suicidality and the interaction effects of CC usage and EC usage on NSSI and suicidality by group (ie, SGM and cis-heterosexual). Both models were controlled for other sociodemographic variables. Results were reported at 95% CIs and were only considered significant when *P* values were less than .05. For variables with a small number of missing values (eg, age, education background), we used mean substitution to input the missing data. We used ω as the measure of the tools’ reliability index [33]. All statistical analyses were performed using SPSS (version 28, IBM Corp).

### Ethics Approval

This cross-sectional survey received approval from the ethics committee at Jilin University (approval 2021-9-29). Informed consent for primary data collection and secondary data analyses from participants was automatically collected from students who selected the commensurate button and continued to answer the questionnaire. The privacy and confidentiality of the participants in this study were protected during data collection and analysis through anonymization and deidentification. None of the personally identifiable information was collected, and all data were stored in a personal computer in the laboratory with password protection. As mentioned earlier, compensation for participants in the study was provided with a link on the postsurvey completion page to the cash reward lottery, with the amount being meticulously calculated to be rewarding while not generating potential bias.

## Results

Table 1 summarizes the sociodemographic characteristics of the participants by group type (ie, SGM or cis-heterosexual). Cigarette usage prevalence was different between SGM participants and cis-heterosexual participants (*χ*^2^_3_[N=89,342]=149.8; *P*<.001). Specifically, the proportion of SGM participants (454/8853, 5.1%) who only used CC was lower than that of their cis-heterosexual counterparts (7161/80,489, 8.9%; *P*<.05); the proportion of SGM participants (53/8853, 0.6%) who only used EC was higher than that of their cis-heterosexual counterpart (348/80,489, 0.4%; *P*<.05). Moreover, the proportion of SGM participants who used CCs was lower than that of their cis-heterosexual counterpart (873/8853, 9.9% vs 10,685/80,489, 13.3%; *χ*^2^_1_[N=89,342]=82.5; *P*<.001, respectively). In contrast, the proportion of SGM participants who used ECs was higher than that of their cis-heterosexual counterpart (472/8853, 5.3% vs 3872/80,489, 4.8%, *χ*^2^_1_[N=89,342]=4.7; *P*=.03, respectively).

Table 2 summarizes the intergroup differences between SGM and cis-heterosexual individuals in this study. The CC dependence score of the SGM group was lower than that of their cis-heterosexual counterpart (*z*=–9.25, *P*<.001). Furthermore, the SGM participants had lower dependence levels on CCs than the cis-heterosexual participants (*χ*^2^_4_[N=89,432]=37.6; *P*<.001). However, compared to the cis-heterosexual group, the SGM group scored higher in EC dependence, suicidality, and NSSI (*z*=3.20, *P*=.001; *z*=69.91, *P*<.001; *z*=54.32, *P*<.001, respectively). At the same time, the SGM group had higher suicide risk and individuals with more NSSI compared to the cis-heterosexual group (*χ*^2^_1_[N=89,432]=4507.5; *P*<.001; *χ*^2^_2_[N=89,432]=3070.9; *P*<.001, respectively). In addition, we analyzed the intergroup difference between the SGM and the cis-heterosexual groups in the revised 6-item scale measuring EC usage. As shown in Table S1 of Multimedia Appendix 1, results from the chi-square test analysis highlighted that compared with the cis-heterosexual group, the SGM group showed a greater degree of EC dependence on each item of the revised scale.

**Table 1:** Sociodemographic and cigarette usage data of the sample population in this study.

Variables			Sexual and gender minority participants (n=8853)		Cis-heterosexual participants (n=80,489)
Age^a^ (years), mean (SD)			19.57 (1.74)		19.60 (1.75)
**Sex assigned at birth, n (%)**					
	Male	2322 (26.2)		35,582 (44.2)	
	Female	6531 (73.8)		44,907 (55.8)	
**Ethnicity, n (%)**					
	Han ethnic group	7850 (88.7)		72,179 (89.7)	
	Others	1003 (11.3)		8310 (10.3)	
**Education background^b^, n (%)**					
	Undergraduate	8361 (94.4)		75,780 (94.2)	
	Masters	477 (5.4)		4516 (5.6)	
	Doctoral	15 (0.2)		193 (0.2)	
**Residence areas prior to university/college enrollment, n (%)**					
	City	5304 (59.9)		40,060 (49.8)	
	Rural/suburban	3549 (40.1)		40,029 (50.2)	
**Only-child status, n (%)**					
	Yes	4506 (50.9)		37,829 (47)	
	No	4347 (49.1)		42,660 (53)	
**Annual family income (¥)^c^, n (%)**					
	<¥6000	2287 (25.8)		24,020 (29.8)	
	¥6000-¥14,000	2831 (32)		26,135 (32.5)	
	¥14,000-¥23,000	1606 (18.1)		13,399 (16.6)	
	¥23,000-¥36,000	948 (10.7)		7894 (9.8)	
	¥36,000-¥70,000	715 (8.1)		5383 (6.7)	
	>¥70,000	466 (5.3)		3658 (4.5)	
**Cigarette usage, n (%)**					
	No	7927 (89.5)		69,456 (86.3)	
	Conventional cigarette	454 (5.1)		7161 (8.9)	
	Electronic cigarette	53 (0.6)		348 (0.4)	
	Dual usage	419 (4.7)		3524 (4.4)	

^a^48 individuals did not answer their age.

^b^13 individuals did not answer their education background.

^c^¥1=US $0.14.

**Table 2:** Intergroup differences between sexual and gender minority and cis-heterosexual participants.^a^

Variables				Sexual and gender minority participants (n=8853)		Cis-heterosexual participants (n=80,489)		*z* score		*χ*^2^ (*df)*			*P* value	
**Conventional cigarettes**														
	Dependance, mean (SD)		0.17 (0.82)		0.24 (0.95)		–9.25		N/A^b^			<.001		
	**Classification, n (%)**										37.6 (4)			<.001
		Very low (0-2)	8590 (97)		77,045 (95.7)									
		Low (3-4)	155 (1.8)		2189 (2.7)									
		Medium (5)	53 (0.6)		607 (0.8)									
		High (6-7)	44 (0.5)		562 (0.7)									
		Very high (8-10)	11 (0.1)		86 (0.1)									
**e-Cigarettes**														
	Dependance, mean (SD)		0.20 (1.21)		0.14 (0.95)		3.20		N/A			.001		
**Suicidality**														
	Mean (SD)		6.19 (3.44)		4.11 (2.12)		69.91		N/A			<.001		
	**Classification, n (%)**										4507.5 (1)			<.001
		Low risk (3-6)	5487 (62)		71,073 (88.3)									
		High risk (7-18)	3366 (38)		9416 (11.7)									
**Nonsuicidal self-injury**														
	Mean (SD)		0.53 (1.20)		0.13 (0.58)		54.32		N/A			.001		
	**Classification, n (%)**										3070.9 (2)			<.001
		Never	6869 (77.6)		75,460 (93.8)									
		Less than 4 days in the past year	1322 (14.9)		3885 (4.8)									
		5 or more days in the past year	662 (7.5)		1144 (1.4)									

^a^The Mann-Whitney *U* test was performed for nonnormally distributed continuous variables (eg, scores) and chi-square test was performed for categorical variables (eg, classifications).

^b^N/A: not applicable.

Table 3 summarizes the effects of cigarette usage and cigarette dependence on NSSI. When only cigarette usage was included in the model (model 1), CC usage, EC usage, and dual usage were associated with increased risks of NSSI when compared to no usage (B=0.12, *P*<.001; B=0.24, *P*<.001; B=0.29, *P*<.001, respectively). However, when cigarette usage and cigarette dependence were both included in the model (model 3), EC usage was not associated with increased risks of NSSI when compared to no usage (B=0.05, *P*=.13). Regardless of whether cigarette dependence was included alone (model 2) or in conjunction with cigarette usage (model 3), EC dependence and CC dependence were associated with increased risks of NSSI (CCs: B=0.04, *P*<.001; B=0.02, *P*<.001; ECs: B=0.06, *P*<.001; B=0.05, *P*<.001, respectively).

**Table 3:** Effects of cigarette usage and dependence on nonsuicidal self-injury.^a^

Variables			Model 1					Model 2					Model 3		
			B (95% CI)		*P* value		B (95% CI)			*P* value		B (95% CI)			*P* value
**Cigarette usage**															
	No cigarette usage	1 (Reference)		Reference		N/A^b^			N/A		1 (Reference)			Reference	
	Conventional cigarette	0.12 (0.10 to 0.13)		<.001		N/A			N/A		0.08 (0.06 to 0.10)			<.001	
	Electronic cigarette	0.24 (0.17 to 0.30)		<.001		N/A			N/A		0.05 (–0.02 to 0.12)			.13	
	Dual usage	0.29 (0.26 to 0.31)		<.001		N/A			N/A		0.10 (0.07 to 0.12)			<.001	
Conventional cigarette dependence			N/A		N/A		0.04 (0.03 to 0.04)			<.001		0.02 (0.02 to 0.03)			<.001
Electronic cigarette dependence			N/A		N/A		0.06 (0.05 to 0.06)			<.001		0.05 (0.04 to 0.06)			<.001

^a^Model adjusted for age, sex assigned at birth, ethnicity, educational background, residence areas prior to university/college enrollment, only-child status, and annual family income.

^b^N/A: not applicable.

Table 4 summarizes the effects of cigarette usage and cigarette dependence on suicidality. When only cigarette usage was included in the model (model 1), CC usage, EC usage, and dual usage were associated with increased risks of suicidality when compared to no usage (B=0.22, *P*<.001; B=0.75, *P*<.001; B=0.82, *P*<.001, respectively). However, when both cigarette usage and cigarette dependence were included in the model (model 3), CC usage was not associated with increased risks of suicidality when compared to no usage (B=0.07, *P*=.07). Regardless of whether cigarette dependence was included alone (model 2) or in conjunction with cigarette usage (model 3), CC dependence and EC dependence were associated with increased risks of suicidality (CCs: B=0.11, *P*<.001; B=0.09, *P*<.001; ECs: B=0.17, *P*<.001; B=0.14, *P*<.001, respectively).

**Table 4:** Effects of cigarette usage and dependence on suicidality.^a^

Variables		Model 1			Model 2			Model 3	
		B (95% CI)	*P* value	B (95% CI)		*P* value	B (95% CI)		*P* value
**Cigarette usage**									
	No cigarette usage	1 (Reference)	Reference	N/A^b^		N/A	1 (Reference)		Reference
	Conventional cigarette	0.22 (0.17 to 0.28)	<.001	N/A		N/A	0.07 (–0.01 to 0.13)		.07
	Electronic cigarette	0.75 (0.52 to 0.98)	<.001	N/A		N/A	0.25 (0.01 to 0.49)		.045
	Dual usage	0.82 (0.74 to 0.89)	<.001	N/A		N/A	0.24 (0.14 to 0.34)		<.001
Conventional cigarette dependence		N/A	N/A	0.11 (0.09 to 0.13)		<.001	0.09 (0.07 to 0.11)		<.001
Electronic cigarette dependence		N/A	N/A	0.17 (0.15 to 0.18)		<.001	0.14 (0.12 to 0.16)		<.001

^a^Model adjusted for age, sex assigned at birth, ethnicity, educational background, residence areas prior to university/college enrollment, only-child status, and annual family income.

^b^N/A: not applicable.

Besides, the multivariable linear regression models showed that cigarette usage has a unique effect on NSSI and suicidality (Table 5). Specifically, the results demonstrated that CC usage, EC usage, and dual usage increased risks for both NSSI and suicidality when compared to no usage (CC: B=0.09, *P*<.001; B=0.22, *P*<.001; EC: B=0.20, *P*<.001; B=0.64, *P*<.001; dual usage: B=0.22, *P*<.001; B=0.68, *P*<.001, respectively). The results also showed that SGM participants had increased risks of NSSI and suicidality when compared to cisgender heterosexual participants (B=0.35, *P*<.001; B=1.94, *P*<.001, respectively). Table 5 underscores the significant interactions of (1) CC usage and group type on NSSI and suicidality (B=0.34, *P*<.001; B=0.24, *P*=.03, respectively) and (2) dual usage and group type on NSSI and suicidality (B=0.54, *P*<.001; B=0.84, *P*<.001, respectively). No significant interaction effect was observed between EC and group type on NSSI and suicidality (B=0.15, *P*=.12; B=0.33, *P*=.32, respectively).

**Table 5:** Interaction effects of cigarette usage with group models.^a^

Variables			Nonsuicidal self-injury					Suicidality		
			B (95% CI)		*P* value		B (95% CI)			*P* value
**Main effects**										
	**Cigarette usage**									
		No	1 (Reference)	Reference		1 (Reference)			Reference	
		Conventional cigarette	0.09 (0.08 to 0.11)	<.001		0.22 (0.16 to 0.28)			<.001	
		Electronic cigarette	0.20 (0.13 to 0.27)	<.001		0.64 (0.40 to 0.88)			<.001	
		Dual usage	0.22 (0.19 to 0.24)	<.001		0.68 (0.60 to 0.75)			<.001	
	Group^b^		0.35 (0.34 to 0.37)	<.001		1.94 (1.89 to 1.99)			<.001	
**Interaction effects**										
	**Cigarette usage × group**									
		Conventional cigarette × group	0.34 (0.28 to 0.41)	<.001		0.24 (0.02 to 0.47)			.03	
		Electronic cigarette × group	0.15 (–0.04 to 0.35)	.12		0.33 (–0.33 to 0.99)			.32	
		Dual usage × group	0.54 (0.47 to 0.61)	<.001		0.84 (0.60 to 1.07)			<.001	

^a^Model adjusted for age, sex assigned at birth, ethnicity, educational background, residence areas prior to university/college enrollment, only-child status, and annual family income.

^b^Group (0=cis-heterosexual participants; 1=sexual and gender minority participants).

In addition, the multivariable linear regression models showed that CC dependence and EC dependence have a unique effect on NSSI and suicidality (Table 6). Specifically, the results demonstrated that participants’ CC and EC dependence simultaneously increased risks for both NSSI and suicidality (CCs: B=0.03, *P*<.001; B=0.12, *P*<.001; ECs: B=0.04, *P*<.001; B=0.13, *P*<.001, respectively). The results also showed that SGM participants had higher levels of NSSI and suicidality when compared to the cisgender heterosexual participants (B=0.38, *P*<.001; B=1.97, *P*<.001, respectively). Table 6 underscores the significant interactions of (1) CC dependence and group type (ie, SGM or cis-heterosexual) on NSSI (B=0.07, *P*<.001) and (2) EC dependence and group type on NSSI and suicidality (B=0.04, *P*<.001; B=0.09, *P*<.001, respectively). No significant interaction effect between CC dependence and group type on suicidality was found (B=–0.01, *P*=.72).

**Table 6:** Interaction effects of cigarette dependence with group models.^a^

Variables		Nonsuicidal self-injury		Suicidality	
		B (95% CI)	*P* value	B (95% CI)	*P* value
**Main effects**					
	Conventional cigarette dependence	0.03 (0.03 to 0.04)	<.001	0.12 (0.10 to 0.13)	<.001
	Electronic cigarette dependence	0.04 (0.04 to 0.05)	<.001	0.13 (0.12 to 0.15)	<.001
	Group^b^	0.38 (0.36 to 0.39)	<.001	1.97 (1.92 to 2.02)	<.001
**Interaction effects**					
	Conventional cigarette dependence × group	0.07 (0.05 to 0.09)	<.001	–0.01 (–0.08 to 0.06)	.72
	Electronic cigarette dependence × Group	0.04 (0.03 to 0.06)	<.001	0.09 (0.04 to 0.14)	<.001

^a^Model adjusted for age, sex assigned at birth, ethnicity, educational background, residence areas prior to university/college enrollment, only-child status, and annual family income.

^b^Group (0=cis-heterosexual participants; 1=sexual and gender minority participants).

## Discussion

### Principal Findings

To our knowledge, this is the first study examining the usage of different cigarette types (ie, CCs and ECs) and their relationship with NSSI and suicidality while investigating intergroup differences between Chinese cis-heterosexual and SGM youth by using data from a large-scale cross-sectional survey. We found that CC usage and dependence were more prevalent among cis-heterosexual individuals, whereas EC usage and dependence were more prevalent among SGM individuals. We found that only CC usage or EC usage increased individuals’ risks for both NSSI and suicidality; in particular, dual usage increased these risks. We also found that CC dependence and EC dependence could simultaneously impact individuals’ risks for NSSI and suicidality independently, and the impacts of each could overlay with the usage of CCs and ECs.

Our findings shed light on the existing intergroup differences in susceptibility to NSSI and suicidality risks. Although CC usage, EC usage, and dual usage increased an individual’s risk of NSSI and suicidality compared with no usage, we found that only CC usage and dual usage increased the risks of NSSI and suicidality in the SGM population compared to those in the cis-heterosexual population. These results are supported by previous research [34-42]. SGM youth are susceptible to the disproportionately high risk of CC usage due to societal, peer, and community influence, which are the major risk factors for NSSI and suicidality. Such societal, peer, and community influences could also be applied to EC usage with its increasing advertisements on social media, and EC usage could be a significant risk factor for mental health risks. Furthermore, recent studies [34-43] underline the fact that there is a significant overlap between CC and EC users in the SGM population, supporting our finding that individuals with dual usage have the highest risks of NSSI and suicidality compared to individuals with no usage of CC and EC.

We found that both CC dependence and EC dependence increased an individual’s risk of NSSI and suicidality. Specifically, we found that both CC and EC dependence increased the risks of NSSI in the SGM population compared to that in the cis-heterosexual population. However, only EC dependence increased the risk of suicidality in the SGM population compared to that in the cis-heterosexual population. Although only a relatively limited amount of research has focused on investigating EC dependence, findings showed that youth with EC usage could experience dependence symptoms (ie, higher usage frequency compared to CC) unique to ECs, creating more barriers to EC cessation [43]. Considering that SGM youth could be more influenced by EC due to societal, peer, and community influence and EC has been deemed as the major risk factor for SGM population’s mental health, we believe our findings on EC dependence and its impact on SGM youth could be valuable for further research. Thus, our findings add to the emerging breadth of evidence emphasizing the imminent risks posed by CC and EC usage and dependence among youth.

The existing intergroup difference between SGM and cis-heterosexual college students in NSSI and suicidality risks resulting from CC and EC usage and dependence can be explained by SGM individuals’ less stable mental health and their vulnerability toward CC usage and EC dependence. First, findings from US and international studies have consistently underlined the continuing worsening situation of SGM youth’s mental health [34-37]. Such a disparity arises from a wide range of structural vulnerabilities such as unavoidable systemic discrimination and prejudice in education, employment, and health care opportunities [38,39]. Further, these structural vulnerabilities stem from the imbalanced hierarchical social power structure that is responsible for generating and widening the mental health gap between SGM and cis-heterosexual individuals while limiting SGM individuals’ accessibility to determinants of health, leaving the SGM population defenseless against risky behaviors such as substance use [39-45].

As previously mentioned, a number of explanations have been proposed in past research, including being impacted by the marketing target of the tobacco industry on the media scale, community norms on a group scale, and minority stressors on a personal scale. First, it is well-established that SGM youth are at disproportionately high risk of CC usage [41,42]. Past research also suggests that the SGM population has been the major marketing target of the tobacco industry, resulting in increased difficulties for SGM youth to resist CC usage when such behavior is normalized by the public media [44]. With the increase in EC usage, recent research underlines that the same aggressive marketing strategy has also been employed by the EC industry and public media. To be more precise, studies indicate that SGM youth were more likely to report being exposed to, searching for, sharing, or being shared with EC–related content on the news or social media platforms compared to their non-SGM peers [46-50]. Second, from a community standpoint, past research has pointed out that the community context could be an influential factor in SGM youth’s smoking behavior [47-50]. Under the influence of increasing exposure to EC-related content among the SGM population, it becomes reasonable to assume that, in social situations with SGM peers (ie, one of the potential triggers for EC usage and combined CC and EC usage), SGM youth would become more susceptible to the rising trend of EC usage among their peers [48]. Third, on a personal scale, as mentioned earlier, the minority stress model underscores that unique stressors experienced by the SGM population, including but not limited to discrimination and internalized homo/bi/transphobia, could result in higher stress levels among this stigmatized group [40-45]. Such stress could then contribute to the thought pattern of EC usage being a way for individuals to express or assert their gender identity or to rebel against traditional gender norms [40-45]. Due to all the aforementioned reasons, concerted efforts need to be made on a societal level in terms of educating SGM youth about the misconceptions, safety concerns, and, most importantly, adverse mental health outcomes such as increased risks for NSSI and suicidality that could be potentially induced by EC usage.

From a broader perspective, the rise of EC usage among Chinese youth has become a public health concern in recent years. Unlike the easy accessibility of the adverse effects of CC usage from various reliable sources, youth often receive inaccurate information about EC usage, resulting in a higher likelihood of maintaining their positive perception of ECs. Under the massive impact created by extensive marketing campaigns, ECs have been promoted as less harmful than CCs and, therefore, have rapidly gained popularity among the younger generation [45]. Compared to the downhill journey of CC usage among Chinese youth (ie, a significant reduction from 12.9% in 2014 to 3.9% in 2019), the rise in EC usage (ie, from 1.2% in 2014 to 2.7% in 2019) reveals that smoking among youth is still a major public health concern in China, many of whom are not fully aware of the physical and mental health risks induced by EC usage [46]. Although camouflaged by the utility for smoking cessation, nonsecondhand smoke exposure, and easy accessibility, ECs’ negative impact on youth’s physical and psychological well-being cannot be ignored. Considering EC’s relationship with NSSI and suicidality, efforts to prevent youth from EC addiction must be made on a societal level [50].

It is worth noting that Chinese government officials and policy makers have promulgated the *Law on Youth Protection* and published the *Notice of the “Guarding Youth” Special Action Plan for Youth Protection from Tobacco Abuse* in 2021 while further strengthening the supervision of EC products, regulating the market order, and standardizing industrial governance by putting into effect the Measures for the Administration of Electronic Cigarettes as of May 1, 2022 [50]. The implementation of these measures has prohibited youth younger than 18 years from purchasing EC products, which could correspondingly reduce their NSSI and suicidality risks. Nevertheless, to prevent the situation from further worsening, future efforts are still required from a societal level to regulate the aggressive marketing of the EC industry and to expand the media and school coverage of educational campaigns in formats such as nonprofit advertisements, school lectures, and short videos on social media platforms on EC usage prevention and its adverse mental health effects on youth.

### Limitations

The findings from this study must be considered in light of a few limitations. First, findings from this study are limited to Chinese SGM college students and, thus, may not be generalizable to SGM youth of other cultural backgrounds or who reside in other countries. Nevertheless, our findings provide crucial insights into the disparity between SGM and cis-heterosexual youth with regard to EC usage, propelling future studies to further investigate the extent and nature of such disparity. Second, although the effect size found in this study was relatively small, our large sample size indicates that such an effect was still stable on a large population scale. Specifically, the impact of cigarette usage and dependence on suicidality and NSSI in SGM youth is more pronounced than that on their cis-heterosexual peers, highlighting the need from a public health standpoint to pay more attention to the impact of EC and CC on suicidality and NSSI among the SGM population. Third, due to the cross-sectional nature of this study, only associations were examined. Longitudinal data are required to investigate these associations over time. Fourth, the sex assigned at birth of the SGM participants in this study was mostly female (6531/8853, 73.8%), which could potentially incur sampling bias and limit the study findings’ generalizability.

### Conclusions and Implications

Overall, our findings have crucial implications for understanding the disparity of NSSI and suicidality risks between SGM and cis-heterosexual youth. First, this study contributes to the growing body of literature highlighting the mental health risks induced by CC usage and EC usage, emphasizing that SGM youth are more susceptible to EC usage. Second, since the sample in this study consisted of Chinese college students, these results could suggest that relevant school policies (eg, CC-free and EC-free campus policies that prohibit EC usage in school public areas and dormitories) and other school-level activities (eg, informative workshops directed by student health centers on EC usage, SGM susceptibility to EC usage, and its physical and mental health risks) could be enacted and hosted to address the health disparity among SGM students. Feedback surveys could be distributed for those policies and school-level interventions to ensure that these interventions attenuate SGM students’ needs rather than perpetuate and exacerbate SGM stereotypical views. Third, findings from this study could inform governmental officials and policy makers on the vulnerability of SGM youth to EC usage. For instance, policy makers could further develop comprehensive EC control policies that are similar to the *Notice of the “Guarding Youth” Special Action Plan for Youth Protection from the Tobacco Abuse* in 2021 but with an emphasis on reducing EC usage and content exposure among SGM youth. Overall, our study provides clear guidance for influential parties to develop effective strategies to resolve the rising imminent public health concern of EC usage among SGM youth.

## Appendix

Multimedia Appendix 1 Electronic cigarette dependence in sexual and gender minority and cis-heterosexual participants.

## Abbreviations

CC: conventional cigarette
EC: electronic cigarette
FTND: Fagerström Test of Nicotine Dependence
NSSI: nonsuicidal self-injury
SBQ-R: Suicidal Behaviors Questionnaire-Revised
SGM: sexual and gender minority
