# Supplementary material for: Effects of Tobacco Versus Electronic Cigarette Usage on Nonsuicidal Self-Injury and Suicidality Among Chinese Youth: Cross-Sectional Self-Report Survey Study
Source: JMIR Public Health Surveill. 2023 Jul 7;9:e47058. doi: 10.2196/47058 (PMC10362422; doi:10.2196/47058)
Supplement: Multimedia Appendix 1 [file publichealth_v9i1e47058_app1.docx]

| **Table S1.** **EC Dependence in SGM and Cis-heterosexual Participants** | | | | | |
| --- | --- | --- | --- | --- | --- |
| Survey Questions | Item options and coding | SGM participants ^a^  (n = 472) | Cis-heterosexual participants ^b^  (n = 3872) | ꭓ^2^ test | *P* |
|  |  | N (%) | N (%) |  |  |
| 1. How soon after your wake up do you smoke your first puff of ECs |  |  |  | ꭓ^2^ = 21.45 | <.001 |
|  | 0=After 2 hours | 213(45.1) | 2067(53.4) |  |  |
|  | 1=60-120 minutes | 50(10.6) | 431(11.1) |  |  |
|  | 2=31-60 minutes | 40(8.5) | 313(8.1) |  |  |
|  | 3=16-30 minutes | 28(5.9) | 251(6.5) |  |  |
|  | 4=6-15 minutes | 52(11.0) | 285(7.4) |  |  |
|  | 5=Within 5 minutes | 89(18.9) | 525(13.6) |  |  |
| 2.Do you find it difficult to refrain from smoking ECs in places where it is forbidden? |  |  |  | ꭓ^2^ = 19.35 | <.001 |
|  | 0=No | 353(74.8) | 3214(83.0) |  |  |
|  | 1=Yes | 119(25.2) | 658(17.0) |  |  |
| 3.Which puff of ECs would you hate to give up? |  |  |  | ꭓ^2^ = 6.17 | .02 |
|  | 0=All the others | 382(80.9) | 3302(85.3) |  |  |
|  | 1=The first one in the morning | 90(19.1) | 570(14.7) |  |  |
| 4. How many times of ECs/day do you smoke? “One time” means 15 puffs or smoking for 10 minutes |  |  |  | ꭓ^2^ = 15.21 | .01 |
|  | 0=0-4 times/day, or 0-60 puffs | 223(47.2) | 2124(54.9) |  |  |
|  | 1=5-9 times/day, or 75-135 puffs | 114(24.2) | 909(23.5) |  |  |
|  | 2=10-14 times/day, or 150-205 puffs | 60(12.7) | 410(10.6) |  |  |
|  | 3=15-19 times/day, or 225-285 puffs | 21(4.4) | 135(3.5) |  |  |
|  | 4=20-29 times/day, or 300-435 puffs | 10(2.1) | 53(1.4) |  |  |
|  | 5=More than 30 times/day, or more than 450 puffs | 44(9.3) | 241(6.2) |  |  |
| 5. Do you smoke ECs more frequently during the first hours after waking than during the rest of the day? |  |  |  | ꭓ^2^ = 8.69 | .004 |
|  | 0=No | 399(84.5) | 3450(89.1) |  |  |
|  | 1=Yes | 73(15.5) | 422(10.9) |  |  |
| 6. Do you smoke ECs if you are so ill you are in bed most of the day? |  |  |  | ꭓ^2^ = 5.65 | .02 |
|  | 0=No | 367(77.8) | 3184(82.2) |  |  |
|  | 1=Yes | 105(22.2) | 688(17.8) |  |  |
| ^a^ 8381 SGM individuals did not answer; ^b^ 80489 Cis-heterosexual individuals did not answer. | | | | | |
